# Associated factors of self-reported psychopathology and health related quality of life among men who have sex with men (MSM) with HIV/AIDS in Dalian, China: a pilot study

**DOI:** 10.1186/s40249-016-0204-z

**Published:** 2016-12-01

**Authors:** Tesfaldet Habtemariam Hidru, Feng Wang, Sainyugu Lolokote, Yong Jia, Min Chen, Wei Tong, Xiao-Feng Li

**Affiliations:** 1 Department of Epidemiology and Biostatistics, Dalian Medical University, Dalian, Liaoning 116044 People’s Republic of China; 2 School of Public Health, Dalian Medical University, Dalian, Liaoning 116044 People’s Republic of China; 3 Department of HIV/AIDS, Dalian Center for Disease Control and Prevention, Dalian, 116023 Liaoning People’s Republic of China

**Keywords:** Men who have sex with men, HIV/AIDS, Psychopathology, Mental health, Health related quality of life

## Abstract

**Background:**

Despite the availability of Anti-Retroviral Therapy (ART), the health-related quality of life (HRQoL) among HIV-positive men who have sex with men (HIVMSM) in China remains poor. This study aimed to explore the relationship between self-reported psychopathology and HRQoL among HIVMSM in Dalian, China.

**Methods:**

A cross-sectional study was conducted in 112 HIVMSM. Symptom check list 90 (SCL 90): a measure of psychopathology and World Health Organization quality of life in HIV infection (WHOQOL-HIV-Bref): a measure of HRQoL were used. Correlation and multiple regression analysis were employed to explore the association between self-reported psychopathology and HRQoL in HIVMSM.

**Results:**

Out of the total 112 HIVMSM cases, 9 (8 %) cases were homosexuals and 103 (92 %) ones were bisexuals. The participants who had low family income (*P* = 0.001), perceived discrimination (*P* = 0.001) and lack of regular attendance in medical follow-ups (*P* = 0.014) reported poorer HRQoL than their counterparts. Somatization, obsession, depression, phobia and overall SCL 90 scores in self-reported psycholpathology had a negative impact on the domains of HRQoL among HIVMSM.

**Conclusions:**

Total quality of life was predicted by family income, perceived discrimination, and medical follow-up attendance. Self-reported psychopathology was negatively correlated with HRQoL in HIVMSM. The Strategies that target MSM focusing on linking and engaging HIV-positive patients in medical care is the key step to improve their HRQoL. More emphasis is needed on the newly diagnosed HIVMSM in Dalian in order to develop a more targeted intervention to prevent perceived discrimination and lack of proper medical follow-up services.

**Trial registration:**

The trial registration number does not require for this manuscript. The study is cross-sectional design that do not involve intervention at all, it is not a clinical trial.

**Electronic supplementary material:**

The online version of this article (doi:10.1186/s40249-016-0204-z) contains supplementary material, which is available to authorized users.

## Multilingual abstracts

Please see Additional file 1 for translations of the abstract into the five official working languages of the United Nations.

## Background

Sexual orientation among men who have sex with men (MSM) is not only culturally and psychologically considered abnormal in China, but also highly discriminated against, as it is considered a high risk for HIV infection. The HIV epidemic and incidence among MSM continues to rise at an alarming rate [1], however the percentage of HIV occurrence in MSM in China used to be relatively low compared to other Asian countries such as Cambodia (7.8 %), Indonesia (9.0 %), and Thailand (24.6 %) [2]. According to the reported statistics in 2002, Chinese MSM represents about 2 to 5 % of the sexually active male population [3]. In recent years, the male-to-male homosexual transmission has become one of the major modes of HIV transmission [4, 5], and the proportion of newly diagnosed HIV cases due to male homosexual contact has increased from 12.2 % in 2007, 21.4 % in 2013, to 23.4 % in 2014, respectively in China [6, 7]. The HIV incidence had increased in Liaoning Province from 5.1 to 10.2 % during 2007–2009 [8], and the HIV infection rate among MSM increased from 4.48 % in 2009 to 12.00 % in Dalian in 2012 [9].

The city of Dalian is in Liaoning province as a peninsula in the Huanghai and the Bohai Sea with an elaborate coastline. It’s beautiful beaches and close proximity to Korea and Japan make it both a domestic and international tourist destination in summer. This flow of people carves and modifies the disposition of local Dalian cultural morays along with the associated sex business with increasing connections via the internet. Although the Chinese society is relatively conservative on sexual issues, the increased number of visitors may impact the culture of the society at large.

The development of highly active antiretroviral therapy (HAART) for HIV/AIDS has helped the patients tremendously in coping with the chronic, complex and unpredictable course of the disease [10–12]. The multiple and complex factors of personality traits [13, 14], age [15] sociocultural stigmatization [16], psychological elements, discrimination and disclosure of HIV/AIDS status pose a substantial challenge to the concept of HRQoL issues among HIV/AIDS infected individuals/patients [17]. Consequently, the aforementioned factors can provoke psychopathological problems and may eventually lead to subnormal quality of health (QoL).

Attitudes towards sex and sexual behaviors have changed throughout eons of cultural revolution in China, however, sexual attitudes towards homosexual engagement are not yet well acceptable and official institutionalization among the gay population is lacking. This is primarily due to the reason that the Chinese society associates a negative connotation to homosexuality and considers it as an aberrant and unacceptable behaviour [18, 19]. Though physical and psychological distress is common amongst the people living with HIV/AIDS [20], the negative implication of the cultural influence and HIV infection might lead to psychosocial instability, which eventually affects the HRQoL of HIVMSM. Also, the emergence of psychopathology and HRQoL is a common observation among the people living with HIV/AIDS [21].

Although a lot of work has been carried out on the risk of HIV/AIDS in the MSM population prior to their infection, far less work has been done to assess the factors that influence the mental health of the HIVMSM post-infection. Until a few years ago, the focus of most researches was on the investigation of HRQoL among the general HIV population and knowledge, attitude and practice towards HIV infection, prevention and test for HIV infection among MSM rather than the mental health wellbeing and HRQoL post-HIV infection. These studies have indicated that socio-demographic characteristics i.e. age, gender, ethnicity, education level, marital status, employment, and transmission route seem to be the primary influential factors affecting the quality of life of the people living with HIV/AIDS [22–24]. Also, the findings from the study related to the quality of life for people living with HIV/AIDS revealed that the factors such as younger age, single, not farmers, and higher education level, high level of CD4 count and good ART adherence tend to have positive effects on QoL [25].

Despite the increased recognition of MSM wellbeing and mental health issues in many parts of China, there is paucity of data on their HRQoL, particularly in Dalian. Also, it is important to consider the alternative foci of research that addresses the post infection mental wellbeing and HRQoL of affected MSM. The present study was designed to assess the self-reported psychopathology and HRQoL for HIVMSM and to explore the associated factors. Since the WHOQOL-HIV- Bref [26] and SCL 90 [27] have proven to be reliable for the assessment of the quality of life and self-reported psychopathology of Chinese people with HIV/AIDS, they were selected to indicate the QoL and self-reported psychopathology in this study. The present study included perceived discrimination, living conditions, and medical follow-up attendance which were not assessed in the earlier studies.

## Methods

A cross-sectional survey of patients aged > 18 years who attended the Center for Disease Control (CDC) in Dalian was conducted. This was a pilot study that targeted the newly diagnosed MSM with HIV/AIDS. All HIVMSM who visited the CDC between December 2012 and December 2013, who were returning to the CDC for antiretroviral treatment follow-up were invited to participate. The patients who were older than 18 years, had sex with men, HIV-infected and exclusively diagnosed in the year of 2012 were eligible for this survey. Patients who had no sexual history with men and were diagnosed earlier or later than 2012, or were transferred from the other parts of China were excluded.

Participants were interviewed using a structured questionnaire to obtain demographic and clinical information, quality of life, and the presence and severity of psychological symptoms. Public health professionals who were trained specifically to collect the data for this study were involved in administering the questionnaires. Newly diagnosed MSM with HIV/AIDS was defined as HIVMSM who had their seropositivity for the first time in year 2012. Income was measured at the household level and classified into 3 groups: low (< 2 000 Yuan), middle (2 000–4 000 Yuan) and high income (> 4 000 Yuan). Fear of discrimination was assessed subjectively by asking the participants about their agreement and disagreement (1 = yes, 2 = no) with a question that assesses if they perceive discrimination from the community or health professionals because of being MSM with HIV/AIDS. Living conditions were determined from the participants’ response (yes or no) to a question whether they are living alone or with family/partner/friend/relative. Medical follow-up attendance was assessed by if the patients regularly attended their follow-up services. Their response recorded 1 = regular or 2 = not regular.

Psychopathology was measured by a Chinese version of SCL-90; a reliable, valid and accepted tool for psychological evaluation [27]. This regimen has been used in several studies in China [28, 29] to explore the presence and severity of psychological symptoms. There are nine subscales of SCL-90 namely; (somatization, obsessive–compulsive, interpersonal sensitivity, depression, anxiety, anger/hostility, phobic anxiety, paranoid ideation and psychoticism). SCL-90 is scored using a five-point scale (0 to 4) to measure the symptoms experienced in the last 7 days. Higher scores indicate more severe psychopathologic symptoms on the SCL- 90.

Participants were appraised for quality of life by WHOQOL-HIV-Bref, a cross-cultural instrument that contains 31-items and six domains. The six domains included in this study were physical, psychological, independence, social relationships, environment, and spirituality, as used in other studies. This self-administered questionnaire explores the issues pertaining to capacity, frequency and intensity or satisfaction. Each item was rated on a five-point Likert scale (1 to 5), with higher scores indicating better functioning. The multidimensional evaluations of the respondents’ health and social life-related conditions were based on how they rate themselves on their daily living activities in all aspects of their lives. The Chinese version of WHOQOL-BREF has been widely used and validated in China [25, 30].

**Fig. 1:**
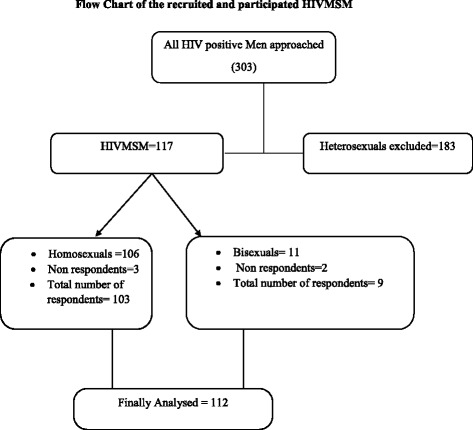
Flow chart of the recruited and participated HIVMSM

A total of 303 HIV-positive men were reported in Dalian in 2012. In the first stage, all HIV-positive men were included in the study. In the second stage, the HIV-positive men were classified into two groups (HIVMSM and heterosexuals) based on their response to their sexual preferences. A total of 117 HIVMSM were identified and included in this study while 186 heterosexuals were excluded from the study. In the third stage, the 117 HIVMSM were classified into homosexuals (*n* = 106) and bisexuals (*n* = 11). All 117 HIVMSM were approached and only 112 (97.5 %) HIVMSM (homosexuals (*n* = 103) and bisexuals (*n* = 9)) participated in this study. Figure 1 demonstrates the flow chart of the recruited and participated HIVMSM.

The data were entered in Epidata 3.1 software to get optimized documentation and error detection, which was further analyzed using IBM SPSS (Statistical Package for Social Sciences), version 21. The differences in psychopathology and HRQoL among the nominal variables of demographic characteristics were tested using independent *t*-tests or one-way analysis of variance (ANOVA). Pearson’s product–moment correlation coefficients were obtained to examine the associations between the continuous variables of demographic characteristics (age and CD4 + T cell count) and, total SCL 90 scores and total HRQoL. The relationship between the self-reported psychopathology (as measured by SCL90) and HRQoL (as measured by the WHO HRQOL-BREF) was also investigated using Pearson’s product moment of correlation. Spearman rank order correlation was used with ranked demographic variables (Marital status, education, family income, employment, living condition, perceived discrimination, sexual behavior and medical follow-up). Multiple regression analyses were conducted to explore the factors that associate with self-reported psychopathology and HRQoL in MSM with HIV/AIDS. The assumptions of normality, independence, linearity and homoscedasticity were checked with a residual analysis. Multicollinearity was assessed for all variables by means of correlation coefficients, tolerances, and the variance inflation factors. Neither assumptions were violated nor was multicollinearity detected. A *P* value of < 0.05 was considered significant.

## Results

### Sociodemographic and clinical characteristics, and self-reported psychopathology and HRQoL

Table 1 shows socio-demographic characteristics of MSM with HIV/AIDS. Out of the total 112 MSM with HIV/AIDS, 9 (8 %) were homosexuals and 103 (92 %) were bisexuals. The mean age of the participants was 34.31 and the mean of their CD4^+^ T cells was 498.5 (SD = 191.6).

**Table 1:** Sociodemographic and clinical characteristics of MSM with HIV/AIDS (*n* = 112)

Demographic and Clinical Characteristics	*n* (%)		
Age		Mean = 34.31	Median = 31
		Range = 18 -73	
Marital status			
Married/co-habiting	16 (14.3)		
Single	78 (69.6)		
Divorced/widowed	18 (16.1)		
Education level			
Junior high school and below	30 (26.8)		
High school or technical secondary school	35 (31.3)		
College and above	47 (42)		
Employment			
Employed	84 (75)		
Nonemployed	28 (25)		
Income			
Low	36 (32.1)		
Moderate	50 (44.6)		
High	26 (23.2)		
Living condition			
With family/partner/friend/relative	79 (70.5)		
Living alone	33 (29.5)		
Discrimination			
Yes	49 (43.7)		
No	63 (56.3)		
Sexual behaviour			
Homosexual	9 (8)		
Bisexual	103 (92)		
CD4^+^T cell count		Mean = 498.5 SD = 191.6 Range = 80–1100	
Medical follow up attendance			
Regular	79 (70.5)		
Irregular	33 (29.5)		

*CD4* ^*+*^ cluster differentiation 4

The participants who perceived discrimination reported significantly higher levels of severity of self-reported psychopathology and poorer HRQoL than those who did not perceive discrimination (*P* < 0.01). Moreover, the participants who reported low family income and lack of regular attendance with their medical follow-ups reported poorer HRQoL than those who had high-income levels and regular medical follow-ups (*P* = 0.001 and *P* = 0.014 respectively). Table 2 shows the differences between sociodemographic and clinical characteristics of MSM with HIV/AIDS in their self-reported psychopathology and HRQoL.

**Table 2:** Differences between sociodemographic and clinical characteristics of MSM with HIV/AIDS, and their self-reported psychopathology and HRQoL

Characteristics of Patients	Self-reported Psychopathology				HRQoL		
	*n* (%)	M (SD)	t/F	*P*	M (SD)	t/F	*P*
Marital status							
Married/co-habiting	16 (14.3)	2.22 (0.81)	2.345	0.101	10.50 (3.14)	0.148	0.862
Single	78 (69.6)	2.24 (0.80)			10.79 (3.13)		
Divorced/widowed	18 (16.1)	1.81 (0.51)			11.11 (3.95)		
Education level							
Junior high school and below	30 (26.8)	2.02 (0.78)	1.153	0.320	10.13 (3.89)	2.132	0.124
Secondary School	35 (31.3)	2.31 (0.83)			10.40 (2.82)		
College and above	47 (42)	2.15 (0.73)			11.53 (3.01)		
Monthly income							
< 2000 RMB	36 (32.1)	2.32 (0.75)	1.127	0.328	9.28 (3.42)	7.857	0.001
2000–4000 RMB	50 (44.6)	2.12 (0.78)			11.12 (2.95)		
> 4000 RMB	26 (23.2)	2.04 (0.79)			12.31 (2.75)		
Employment							
Yes	84 (75)	2.11 (0.76)	−1.307	0.194	11.00 (3.13)	1.109	0.270
No	28 (25)	2.33 (0.82)			10.21 (3.58)		
Living condition							
Alone	79 (70.5)	2.12 (0.73)	−1.024	0.308	10.81 (3.32)	0.033	0.974
Co-resident	33 (29.5)	2.28 (0.86)			10.79 (3.12)		
Discrimination							
Yes	49 (43.7)	2.44 (0.80)	3.434	0.001	8.82 (2.45)	−6.762	0.000
No	63 (56.3)	1.95 (0.69)			12.35 (2.95)		
Sexual behaviour							
Homosexual	9 (8)	2.14 (0.78)	−0.124	0.901	10.67 (3.16)	−0.131	0.896
Bisexual	103 (92)	2.17 (0.78)			10.82 (3.27)		
Medical follow ups							
Regular	79 (70.5)	2.17 (0.78)	0.341	0.734	11.29 (3.06)	2.494	0.014
Irregular	33 (29.5)	2.12 (0.75)			9.63 (3.50)		

*Note*: The number and percentage of the participants in HRQoL are equivalent to the *n* and % in Psychopathology

### Determinants of HRQoL

- **Psychopathology**

Table 3 illustrates the correlation between sociodemographic characteristics and psychopathologic symptoms among MSM with HIV/AIDS. For MSM with HIV/AIDS, SCL 90 scores were significantly correlated with fear of discrimination (*P* < 0.01).

**Table 3:** Self-reported psychopathology among MSM with HIV/AIDS

	SCL-90 domains									
Sociodemographic characteristics	Somatization	Obsession	Interpersonal	Depression	Anxiety	Hostility	Phobia	Paranoid	Psychotic	Total score
Age	-.044	-.030	.007	-.051	-.071	-.099	-.072	-.096	-.042	-.044
Marital Status	-.125	-.126	-.165	-.142	-.147	-.126	-.160	-.163	-.212*	-.157
Education	.054	.056	.035	.076	.019	-.040	.022	.098	.050	.054
Family Income	-.145	-.153	-.152	-.075	-.004	-.185	-.155	-.122	-.138	-.145
Employment	.078	.187*	.123	.106	.081	.075	.113	.122	.093	.129
Living condition	.039	.134	-.026	.082	.093	.166	.041	.106	.030	.072
Perceived discrimination	-.289**	-.360**	-.261**	-.287**	-.257**	-.217*	-.315**	-.217*	-.274**	-.320**
Sexual behaviour	.018	-.023	-.056	.053	-.001	.014	-.066	-.049	.025	.005
CD4 + T cell count	-.045	.114	.009	-.053	.084	.001	.061	.017	.018	-.045
Medical follow up	-.081	-.021	.048	.023	-.035	-.042	.036	.062	.015	-.011

*Notes*: *P < 0.05; **P < 0.01

b. **HRQoL**

Table 4 shows the correlation of HRQoL among MSM with HIV/AIDS. Family income (*P* < 0.01), fear of discrimination (*P* < 0.01) and medical follow-up attendance (*P* < 0.05) were significantly correlated with WHO-HIV-Bref-QOL domains among MSM with HIV/AIDS. In addition, subscales of SCL 90 (somatization, obsession, depression and phobia) and total scores of SCL 90 were found to be correlated with total scores of HRQoL.

**Table 4:** Health-related quality of life among MSM with HIV/AIDS

		WHO-HIV-Bref-QOL domains						
		Physical	Psychological	Independence	Social	Environment	Spirituality	Total QoL
Sociodemographic characteristics	Age	.072	.031	-.056	-.057	.020	.192*	-.017
	Marital Status	.105	.022	-.034	-.048	.046	.125	.007
	Education	.048	.065	.143	.114	.136	.033	.184
	Family income	.256**	.306**	.352**	.284**	.357**	.298**	.351**
	Employment	-.111	-.111	-.049	-.031	-.120	-.199*	-.092
	Living condition	.012	.097	.006	.107	-.015	.087	-.015
	Perceived discrimination	.305**	.200*	.352**	.402**	.409**	.276**	.570**
	Sexual behavior	-.004	-.038	.056	.055	.034	.018	.044
	CD4 + T cell count	.050	.147	.026	.168	.038	.112	.053
	Medical follow up	-.233*	-.280**	-.125	-.116	-.143	-.160	-.233*
SCL-90	Somatization	-.223*	-.134	-.258**	-.140	-.258**	-.138	-.230*
	Obsession	-.264**	-.181	-.177	-.149	-.232*	-.260**	-.257**
	Interpersonal	-.181	-.130	-.107	-.088	-.174	-.225*	-.152
	Depression	-.208*	-.210*	-.140	-.089	-.183	-.213*	-.197*
	Anxiety	-.205*	-.155	-.134	-.080	-.157	-.193*	-.169
	Hostility	-.041	-.004	-.015	.053	-.035	-.077	-.116
	Phobia	-.233*	-.195*	-.211*	-.094	-.230*	-.268**	-.273**
	Paranoid	-.147	-.121	-.139	-.039	-.163	-.136	-.179
	Psychotic	-.173	-.168	-.108	-.042	-.168	-.193*	-.155
	Total SCL-90 score	-.216*	-.167	-.164	-.098	-.204*	-.212*	-.212*

*Notes*: *P < 0.05; **P < 0.01

c. **Predictors of self-reported psychopathology**

Total SCL 90 score explained 8.4 % of variance (*R2* = 0.084). All measures of psychopathology were predicted by the perception of discrimination. Interpersonal sensitivity was also predicted by marital status (β = −0.251; *P* = 0.041). Table 5 describes the predictors of self-reported psychopathology among MSM with HIV/AIDS.

**Table 5:** Predictors of self-reported psychopathology among MSM with HIV/AIDS

Subscales	Item	β	T	*P*	Γ^2^	95 % *CI*
Somatization	Perceived discrimination	−0.308	−3.118	0.002	0.048	−0.880 ~ −0.196
Obsession	Perceived discrimination	−0.327	−3.419	0.001	0.111	−0.887 ~ −0.236
Interpersonal sensitivity	Marital status	−0.251	−2.065	0.041	0.049	−0.553 ~ −0.011
	Perceived discrimination	−0.214	−2.164	0.033		−0.699 ~ −0.030
Depression	Perceived discrimination	−0.224	−2.250	0.027	0.035	−0.801 ~ −0.050
Anxiety	Perceived discrimination	−0.250	−2.520	0.013	0.037	−0.812 ~ −0.097
Hostility	Perceived discrimination	−0.225	−2.253	0.026	0.029	−0.670 ~ −0.043
Phobia	Perceived discrimination	−0.254	−2.594	0.011	0.066	−0.752 ~ −0.100
Psychotic	Perceived discrimination	−0.238	−2.414	0.018	0.050	−0.684 ~ −0.067
Total SCL 90 scores	Perceived discrimination	−0.282	−2.881	0.005	0.065	−0.740 ~ −0.137

d. **Predictors of health-related quality of life among MSM with HIV/AIDS**

All domains of HRQoL, except social domain, were predicted by family income. Social and spirituality domains, and total HRQoL were predicted by fear of discrimination (β = 0.404; *P* < 0.001, β = 0.218; *P* = 0.049 and β = 0.433; *P* < 0.001 respectively). Medical follow-up attendance seems to be a significant predictor of physical, psychological and social domains, and total HRQoL. Table 6 illustrates the predictors of health-related quality of life among MSM with HIV/AIDS.

**Table 6:** Predictors of health-related quality of life among MSM with HIV/AIDS

Domain	Item	β	T	*P*	Γ^2^	95 % *CI*
Physical	Marital status	0.307	2.269	0.026	0.184	0.160 ~ 2.472
	Family income	0.236	2.115	0.038		0.062 ~ 2.073
	Medical follow-up	−0.211	−2.045	0.045		−3.148 ~ −0.040
Psychological	Monthly family income	0.319	2.901	0.005	0.200	0.405 ~ 2.181
	Medical follow-up	−0.342	−3.372	0.001		−3.675 ~ −.945
Independence	Monthly family income	0.332	3.111	0.003	0.244	0.553 ~ 2.522
Social	Monthly family income	0.266	2.629	0.010	0.328	0.285 ~ 2.070
	Living condition	0.256	2.742	0.008		0.522 ~ 3.301
	Perceived discrimination	0.404	4.088	0.000		1.411 ~ 4.095
	Medical follow-up	−0.194	−2.067	0.042		−2.810 ~ −0.051
Environment	Monthly family income	0.290	2.631	0.010	0.192	0.286 ~ 2.073
Spirituality	Monthly family income	0.265	2.373	0.020	0.185	0.208 ~ 2.395
	Perceived discrimination	0.218	2.004	0.049		0.009 ~ 3.296
Total HRQoL	Monthly family income	0.231	2.359	0.021	0.364	0.159 ~ 1.890
	Perceived discrimination	0.433	4.521	0.000		1.649 ~ 4.247
	Medical follow-up	−0.225	−2.488	0.015		−2.991 ~ −0.331

## Discussion

This study presents prevalent bisexual preferences among HIVMSM in Dalian. The findings from this study demonstrate that 92 % of the HIVMSM were bisexuals while only 8 % were homosexuals. Culturally, homosexuality is an unacceptable behaviour in China [19], which could be a reason for the high bisexual preference to cover up and decrease the pressure from the society.

In this study, MSM who reported the perception of discrimination had a higher level of severity of psychopathology (*P* = 0.001) and poor HRQoL (*P* < 0.001). This could be attributed to internalization of negative attitudes and assumptions of guilt, inferiority and lack of self-worth [31], which may lead to self-blame and self-isolation due to perceived discrimination, probably exacerbating hostility to social environments. Evidence from a published report revealed that a hostile social environment has an impact on mental health and QOL as homosexuals reported higher levels of sexual minority-specific victimization, depressive symptoms, and suicidality compared to the heterosexuals [32].

A survey from Jinan, Qingdao, and Yantai of Shandong province in China concluded that the bisexual behaviour is independently associated with higher levels of HIV/AIDS-related discrimination [33]. Social (*P* < 0.001) and spirituality (*P* = 0.049) domains, and total HRQoL (*P* < 0.001) were predicted by perceived discrimination. On the other hand, self-reported symptoms; somatization (*P* < 0.05), obsession (*P* < 0.01), depression (*P* < 0.05), phobia (*P* < 0.01) and overall SCL 90 scores (*P* < 0.05) were significantly correlated with total quality of life (TQOL). A similar study among the individuals living with HIV/AIDS reported significant associations between poor HRQoL and a high degree of depression, anxiety, anger and low self-confidence [13]. Homosexuality is taboo in China, and therefore, MSM often feel guilt, low self-esteem, and fear of discrimination, which may eventually lead to depression and decreased HRQoL [34, 35]. The negative impact of depression in QOL among people living with HIV/AIDS and its role in disease progression has been described before [36, 37]. The present study shows that all subscales of SCL 90 including total SCL 90 score were predicted by perceived discrimination. The self-reported psychopathology due to the experience of perceived discrimination may affect the HRQoL of the HIVMSM through: (i) impairing their self-esteem and self-confidence, (ii) worsening their stress, (iii) increasing unemployment that can affect their income level, (iv) increasing social withdrawal that can result in loneliness and poor medical follow-up attendance, and (v) decreasing their motivation that may result in self-care deficit and poor self-image. The poor HRQoL observed among HIVMSM in this study could benefit from psychological and social support.

Lack of proper medical follow-up emerged as a significant predictor of physical (*P* = 0.045), psychological (*P* = 0.001), social domains (*P* = 0.042) and total HRQoL (*P* = 0.015) in this study. Medical check-ups provide a better opportunity to the people living with HIV/AIDS to communicate with the health professional on their medications and any concerns regarding their physical and mental health. One qualitative study in Zambia identified lack of follow-up and counselling as a barrier to patient’s adherence to ART [38]. Hence, follow-up and counselling should be strengthened to provide the information about ART and better comprehensive medical check-ups.

The persistent influence of family income in HRQoL between HIVMSM observed in this study and ordinary HIV/AIDS cases from the previous studies might show the exposure of the same influential or risk factors that are associated with socioeconomic status. High family income [39] and unemployment [15] have positive and negative associations respectively with QOL among people living with HIV/AIDS. Furthermore, economic satisfaction and family support were reported among the conditions that can positively influence HRQoL among the adults with HIV/AIDS [40].

The findings from our study provide primary predictors (perceived discrimination, low-income, and irregular medical follow-up) that are associated with psychopathology and poor HRQoL, and the corresponding proportions of the sexual behaviours among the MSM with HIV/AIDS. The understanding of the correlations and predictors that may impact the mental wellbeing and quality of life among the HIVMSM is essential for improving the continuum of healthcare plan for MSM in Dalian. This will foster the accessibility of HIVMSM to HIV patient care and consequently contribute to the healthcare policy development regarding the prevention and intervention. HIV transmission among MSM is surging in China and our current study revealed significantly high psychopathological symptoms and poor HRQoL among HIVMSM who have reported perceived discrimination.

This study has several limitations. First, this study is subjected to self-reported data. Second, there was no confirmatory assessment that can prove the presence or severity of psychopathology, and the magnitude of the perception of discrimination in addition to the participants’ report. The strength of this study lies in its broad representative samples for the recorded HIVMSM and the use of a standardized instrument.

## Conclusion

According to this study total quality of life was predicted by family income, perceived discrimination and medical follow-up attendance whereas self-reported psychopathology was predicted by perceived discrimination. To reduce the psychopathologic symptoms among HIVMSM, social and psychological support is of crucial importance to improve HRQoL in this targeted population. More needs to be emphasized on the newly diagnosed HIVMSM in Dalian in order to develop a more targeted intervention to prevent perceived discrimination and lack of proper medical follow-up services. The strategies targeting MSM and focusing on linking and engaging HIV-positive patients in a healthcare are the keys to bridging the steps to improving HIV healthcare. This can be accomplished by establishing free HIV care, advocacy for MSM with HIV-positive by health professionals, and extending community health education to avoid stigma and discrimination from the society in order to improve the mental well-being and health-related quality of life.

## Additional file

Additional file 1: Multilingual abstracts in the five official working languages of the United Nations. (PDF 738 kb)

## Abbreviations

AIDS: Acquired immunodeficiency syndrome
ART: Anti-retroviral therapy
CD4: Cluster differentiation 4
HAART: Highly active anti-retroviral therapy
HIV: Human immunodeficiency virus
HRQoL: Health related quality of life
MSM: Men who have sex with men
QOL: Quality of life
SCL 90: Symptom check list 90
WHO: World Health Organization
WHOQOL-HIV-Bref: World Health Organization quality of life in HIV infection
