# Supplementary material for: Associated factors of self-reported psychopathology and health related quality of life among men who have sex with men (MSM) with HIV/AIDS in Dalian, China: a pilot study
Source: Infect Dis Poverty. 2016 Dec 1;5:108. doi: 10.1186/s40249-016-0204-z (PMC5131411; doi:10.1186/s40249-016-0204-z)
Supplement: Additional file 1: — Multilingual abstracts in the five official working languages of the United Nations. (PDF 738 kb) [file 40249_2016_204_MOESM1_ESM.pdf]

Translation of the abstract into the five official working languages of the United Nations

العوامل المرتبطة بالأمراض النفسية المبلّغ عنها ذاتيا و طبيعة الحياة الصحية للرجال الذين أقامو علاقة جنسية مع رجال مصابين بمرض نقص المناعة المكتسبة في داليان في الصين: دراسة استطلاعية

تسفالديت هابفيماريام هيدرو- فينغ وانغ- سينيوغو لولوكوت- يونغ جيا- مين تشين- وي تونغ- تشيوفينغ مي.

ملخص الدراسة

خلفية الموضوع

على الرغم من توافر العلاج بمضادات الفيروسات القهقرية إلا أن طبيعة الحياة الصحية لاتزال متدنية بين الرجال الحاملين لفيروس نقص المناعة المكتسبة اللذين يمارسون الجنس مع الرجال. تهدف هذه الدراسة الى استكشاف العلاقة بين الأمراض النفسية المبلّغ عنها ذاتيا وطبيعة الحياة الصحية للرجال الحاملين لفيروس نقص المناعة المكتسبة اللذين يمارسون الجنس مع الرجال في مدينة داليان في الصين.

المنهجية

تم إجراء دراسة مستعرضة ل 112 حالة لرجال حاملين لفيروس نقص المناعة المكتسبة و يمارسون الجنس مع الرجال. قائمة مراجعة الأعراض ال 90: مقياس المرض النفسي و طبيعة الحياة الصحية لعدوى مرض نقص المناعة المكتسبة كما تم توضيحها من قبل منظمة الصحة العالمية: تم استخدام مقياس طبيعة الحياة الصحية. تم استخدام تحليل الإرتباط وتحليل الانحدار المتعدد لاستكشاف العلاقة بين الأمراض النفسية المبلّغ عنها ذاتيا و طبيعة الحياة الصحية لدى الرجال المصابين بمرض نقص المناعة المكتسبة الذين يمارسون الجنس مع الرجال.

النتائج: تبين من التائج أن 9 حالات (8%) من مجمل الحالات ال 112 كانو مثليي الجنس وتبين أن 103 (92%) من الحالات كانو ثنائيي الجنس. وقد أظهرت النتائج أيضا أن المشاركين ذوي الدخل المنخفض (p=0.001) والذين يتعرضون للتميز (p=0.001) وعدم الإنتظام في حضور جلسات المتابعة الطبية (p=0.014) لديهم طبيعة حياة صحية متدنية مقارنة مع نظرائهم. فقد تبين أن لاضطراب الجسدنة والهوس والإكتئاب والرهاب وإجمالي قائمة مراجعة الأعراض ال 90 في حالات المرض النفسي الذي تم إبلاغ عنه ذاتيا قد أثّر سلبياً على مجالات طبيعة الحياة الصحية للرجال المصابين بمرض نقص المناعة الكتسبة الذين يمارسون الجنس مع الرجال.

الخاتمة: تم التنبؤ بطبيعة الحياة بشكل عام من خلال دخل الأسرة والتمييز المحسوس وحضور جلسات المتابعة الطبية. فقد ارتبط المرض النفسي المبلّغ عنه ذاتيا بطبيعة الحياة الصحية لدى الرجال الحاملين لفيروس نقص المناعة المكتسبة الذين يمارسون الجنس مع الرجال. فالاستراتيجيات التي تستهدف الرجال الذين يمارسون الجنس مع الرجال تركز على ربط واشراك المرضى الحاملين لفيروس الايدز في الرعاية الصحية وهي الخطوة الأساسية لتحسين طبيعة الحياة الصحية. وهناك حاجة إلى مزيد من التركيز على الحالات التي يتم تشخيصها حديثاً للرجال المصابين بنقص المناعة المكتسبة ويمارسون الجنس مع الرجال في داليان ليتم تطوير تدخل أكثر فعالية لمنع التميزالذي يمكن التنبؤ به ونقص خدمات المتابعة الصحية المناسبة.

Translated from English version into Arabic by Randa82, through

TRANSLATORS
WITHOUT BORDERS

HIV/AIDS 感染的男男性接触者症状自评心理状态和健康相关生活质量及其影响因素的研究----中国大连地区的一项初步研究

Tesfaldet Habtemariam Hidru, 王丰, Sainyugu Lolokote, 贾勇, 陈敏, 佟伟, 李晓枫

摘要

引言：尽管已提供了免费的抗逆转录病毒疗法(ART)，中国 HIV 阳性的男男性接触者(HIVMSM)的健康相关生活质量(HRQoL)仍然是较低的。本研究旨在初步探讨中国大连地区 HIVMSM 者的症状自评心理状态与健康相关生活质量(HRQoL)的关系及其影响因素。

方法：本研究采用横断面研究的方法，随机抽取 112 名 HIV 阳性的男男性接触者作为研究对象。应用 SCL-90 量表测量精神、心理健康状态，同时使用世界卫生组织艾滋病生存质量测量简表(WHOQOL-HIV-Bref)测量健康相关生活质量（HRQoL）。统计分析方法采用相关分析和多元回归分析。

结果：在 112 个 HIVMSM 患者中，有 9 例同性恋(占 8%)和 103 例双性恋(占 92%)。研究结果表明，家庭收入低(P=0.001)、感觉受到歧视(P=0001)、没有定期参加医学随访者(P=0.014)

的健康相关生活质量更低。SCL-90 总分及躯体化、强迫、抑郁和恐怖因子得分与 HRQoL 的各领域得分呈负相关。
结论：健康相关生活质量可由家庭收入、是否感觉受到歧视以及是否定期参加医学随访这些因素来预测。HIVMSM 患者的 SCL-90 得分与 HRQoL 呈负相关。制订有针对性的预防措施是提高男男性接触者健康相关生活质量的关键。在大连地区，对于新诊断的 HIVMSM 患者，应采取更有针对性的预防措施以避免歧视和提供更合适的医学随访服务。

Translated from English version into Chinese by Xiao-Feng Li

Facteurs associés à la psychopathologie et à la qualité de vie liée à la santé chez les hommes séropositifs ayant des rapports sexuels avec d'autres hommes (HSH) dans la ville de Dalian, en Chine : étude pilote

Tesfaldet Habtemariam Hidru, Feng Wang, Sainyugu Lolokote, Yong Jia, Min Chen, Wei Tong, Xiao-Feng Li

RÉSUMÉ

Contexte : en Chine, la qualité de vie liée à la santé (QVLS) des hommes séropositifs ayant des rapports sexuels avec d'autres hommes (HSH séropositifs) reste précaire, et ce, malgré l'accès aux traitements antirétroviraux. Cette étude avait pour but de découvrir le lien entre la psychopathologie et la QVLS des HSH séropositifs dans la ville de Dalian, en Chine.
Méthodes : une étude transversale a été menée sur 112 HSH séropositifs. Un questionnaire permettant l'évaluation du profil psychopathologique, le SCL-90-R (questionnaire de santé mentale), et une mesure de la QVLS (l'outil de l'Organisation mondiale de la santé mesurant la qualité de vie des personnes séropositives) ont été utilisés. La corrélation et l'analyse de régression multiple ont été utilisées pour déterminer le lien entre la psychopathologie et la QVLS des HSH séropositifs.
Résultats : sur les 112 HSH séropositifs interrogés, 9 étaient homosexuels (soit 8 %) et 103 étaient bisexuels (soit 92 %). Il apparaît que les sujets ayant un revenu familial faible (*P*=0,001), ayant ressenti de la discrimination (*P*=0,001) et ne s'étant pas présentés régulièrement aux suivis médicaux (*P*=0,014) ont une QVLS plus précaire que les autres participants. La somatisation, l'obsession-compulsion, la dépression, l'anxiété phobique et, de façon générale, les réponses du SCL-90-R sur la psychopathologie, ont un impact négatif sur la QVLS des HSH séropositifs.
Conclusions : le revenu familial, la discrimination perçue et la présence aux suivis médicaux réguliers influent sur la qualité de vie. Aucun lien n'a été déterminé entre la psychopathologie en elle-même et la QVLS des HSH séropositifs. Les stratégies visant à encourager les HSH vivants avec le VIH à être pris en charge au niveau médical sont une étape clé dans le processus d'amélioration de la QVLS des sujets. L'attention portée aux HSH de Dalian récemment diagnostiqués séropositifs doit être plus importante encore. Cette intervention ciblée permettra de mieux prévenir la discrimination et d'encourager les suivis médicaux réguliers.

Translated from English version into French by Alexandra Das Neves, through

TRANSLATORS WITHOUT BORDERS

Связанные факторы автоматической психопотологии и связанное с состоянием здоровья качество жизни мужчины с ВИЧ/СПИД, который занимается сексом с мужчиной(МСМ), в городе Даляне, Китае: пионерное изучение

Tesfaldet Habtemariam Hidru, Feng Wang, Sainyugu Lolokote, Yong Jia, Min Chen, Wei Tong, Xiaofeng Li

РЕФЕРАТ:
Фон: не смотря на доступность антиретровирусной терапии, бедным остаётся связанное с состоянием здоровья качество жизни китайского мужчины, который занимается сексом с мужчиной. Цель этого исследования —изучать отношения между автоматической психопотологией и связанным с состоянием здоровья качеством жизни МСМ с ВИЧ в Даляне, Китае.
Методы: Было выполнено типичное исследование 112 МСМ с ВИЧ. Применили опросник выраженности психопатологической симптоматики(SCL90): измерение, применяемое в психопатологии, и качества жизни человека ВИЧ-инфицированного, измерение связанного с состоянием здоровья качества жизни, применяемое Всемирной организацией здравоохранения. Коррелятивный и многократный регрессионный анализ провели , чтобы изучать отношения между автоматической психопатологией и связанным с состоянием здоровья качеством жизни МСМ с ВИЧ.
Результаты: 9(8%) из 112 респондентов были гомосексуальными и остальные 103 (92%) - бисексуальными. Как наблюдели, было хуже чем у других связанное с состоянием здоровья качество жизни у тех участников , у которых был ниже семейный доход（P=0.001）, ощутимая дискриминация (P=0.001) и недостаток регулярного медицинского наблюдения (P=0.014). Соматизация, одержимость, депрессия, фобия оказывают негативное влияние на связанное с состоянием здоровья качество жизни МСМ с ВИЧ.
Заключения: По семейному доходу, ощутимой дискриминации и медицинскому наблюдению было предсказано качество жизни. Автоматическая психопатология не положительно влияет на связанное с состоянием здоровья качество жизни МСМ с ВИЧ. Стратедия, которая стремится соединять ВИЧ-положительных больных и привлекать их к медицинской помощи, является ключевым шагом для улучшения связанного с состоянием здоровья качества их жизни. Надо обращать больше внимания на МСМ недавно поставлены диагноз ВИЧ-инфекции, чтобы развивать более целенаправленное вмешательство, предотвращающее ощутимую дискриминацию и отсутсвие подходящей медицинской услуги.

Translated from English version into Russian by Hao-Qi Zhang

Factores asociados de psicopatología y calidad de vida relacionada con la salud autodeclaradas entre hombres que mantienen relaciones sexuales con hombres con VIH/sida en Dalian, China: estudio piloto

Tesfaldet Habtemariam Hidru, Feng Wang, Sainyugu Lolokote, Yong Jia, Min Chen, Wei Tong, Xiaofeng Li

ABSTRACT
Antecedentes: A pesar de la disponibilidad de terapia antirretrovírica (ART, por sus siglas en inglés), la calidad de vida relacionada con la salud (HRQoL) continúa siendo mala entre los hombres VIH seropositivos que mantienen relaciones sexuales con hombres (HIVMSM) en China. Este estudio tiene como objetivo explorar la relación entre la psicopatología y la HRQoL autodeclaradas entre HIVMSM en Dalian, China.
Métodos: Se llevó a cabo un estudio transversal en 112 HIVMSM. Se utilizó el Cuestionario de Síntomas 90 (SCL 90) como medición de psicopatología y el WHOQOL-HIV-Bref sobre calidad de vida en la infección por el virus del VIH de acuerdo con la Organización Mundial de la Salud como medición de la HRQoL. Para explorar la asociación entre la psicopatología y la HRQoL autodeclaradas en los HIVMSM se empleó un análisis de regresión y correlación múltiple.
Resultados: Del total de 112 casos de HIVMSM, 9 (el 8%) eran homosexuales y 103 (el 92%) eran bisexuales. Los participantes que tenían ingresos familiares bajos (P=0.001), que percibieron discriminación (P=0.001) y que no asistieron regularmente a los controles médicos (P=0.014) declararon peor HRQoL que sus contrapartes. La somatización, obsesión, depresión, fobia y las puntuaciones generales del SCL 90 tuvieron un impacto negativo en los dominios de HRQoL entre los HIVMSM.

Conclusiones: La calidad de vida total se predijo mediante el ingreso familiar, la discriminación percibida y la asistencia a los controles médicos. La psicopatología autodeclarada se correlacionó negativamente con la HRQoL en los HIVMSM. Las estrategias que apuntan a los MSM concentrándose en comunicarse e involucrar a los pacientes VIH-positivos en la atención médica es el paso clave para mejorar su HRQoL. Es preciso hacer más hincapié en los HIVMSM recientemente diagnosticados de modo tal de desarrollar una intervención más dirigida para prevenir la discriminación percibida y la falta de servicios de control médico adecuados.

Translated from English version into Spanish by Marisa Condurso de Nohara, through

TRANSLATORS
WITHOUT BORDERS
